# Retroperitoneal leiomyosarcoma in a female patient with a germline splicing variant *RAD51D* c.904-2A > T: a case report

**DOI:** 10.1186/s13053-021-00205-x

**Published:** 2021-11-27

**Authors:** Mashu Futagawa, Hideki Yamamoto, Mariko Kochi, Yusaku Urakawa, Reimi Sogawa, Fumino Kato, Mika Okazawa-Sakai, Daisuke Ennishi, Katsunori Shinozaki, Hirofumi Inoue, Hiroyuki Yanai, Akira Hirasawa

**Affiliations:** 1 Department of Clinical Genomic Medicine, Graduate School of Medicine, Dentistry and Pharmaceutical Sciences, Okayama University, 2-5-1 Shikata-cho, Kita-ku, Okayama, 700-8551 Japan; 2 Department of Clinical Genomic Medicine, Okayama University Hospital, Okayama, Japan; 3 Center for Comprehensive Genomic Medicine, Okayama University Hospital, Okayama, Japan; 4 Division of Clinical Oncology, Hiroshima Prefecture Hospital, Hiroshima, Japan; 5 Department of Pathology, Okayama University Hospital, Okayama, Japan

**Keywords:** *RAD51D*, Splice variant, Leiomyosarcoma, Homologous recombination (HR), Cancer susceptibility, Presumed germline pathogenic variant (PGPV)

## Abstract

**Background:**

*RAD51D* (RAD51 paralog D) is an intermediate cancer susceptibility gene for primary ovarian cancer, including fallopian tube and peritoneal carcinomas and breast cancer. Although gynecological non-epithelial tumors such as uterine sarcomas are associated with genomic instability, including *BRCA* impairment, there is no clear evidence of the relationship between *RAD51D* variants and the risk of sarcoma development.

**Case presentation:**

A Japanese woman in her 50s underwent multiple surgical resections and several regimens of chemotherapy for tumors that originated in the retroperitoneum and recurred in the peritoneum over a clinical course of approximately 4 years. The peritoneal tumor was histologically diagnosed as a leiomyosarcoma and was genetically identified to show a splice variant of *RAD51D* c.904-2A > T [NM_002878] through tumor profiling performed as a part of cancer precision medicine. The confirmatory genetic test performed after genetic counseling revealed that the *RAD51D* splicing variant detected in her tumor was of germline origin. In silico analyses supported the possible pathogenicity of the detected splice variant of *RAD51D* with a predicted attenuation in mRNA transcription and truncated protein production due to frameshifting, which was attributed to a single-nucleotide alteration in the splicing acceptor site at the 3′-end of intron 9 of *RAD51D*. Considering her unfavorable clinical outcome, which showed a highly aggressive phenotype of leiomyosarcoma with altered *RAD51D*, this case provided novel evidence for the relationship of a *RAD51D* splicing variant with malignant tumor development or progression. We report the findings of this rare case with possible involvement of the germline variant of *RAD51D* c.904-2A > T as a potential predisposing factor for malignant tumors, including leiomyosarcoma.

**Conclusions:**

We present the findings of a case of leiomyosarcoma in the peritoneum of a female patient with a novel germline splicing variant of *RAD51D* as potential evidence for the pathogenicity of the variant and its involvement in the risk of sarcoma etiology and/or development. To the best of our knowledge, this is the first case report describing a leiomyosarcoma carrying a germline *RAD51D* splicing variant and elucidating its pathogenicity on the basis of computational prediction of the impairment of normal transcription and the presumed loss of functional protein production.

**Supplementary Information:**

The online version contains supplementary material available at 10.1186/s13053-021-00205-x.

## Background

The RAD51 paralog D (RAD51D) is one of five RAD51 paralog members, namely, RAD51B (RAD51L1), RAD51C (RAD51L2), RAD51D (RAD51L3), XRCC2, and XRCC3. These members assist in the recruitment of RAD51, which functions as a DNA recombinase during DNA replication and plays crucial roles in DNA double-strand break (DSB) repair by homologous recombination (HR) on damaged DNA [1, 2]. RAD51D forms a subcomplex with RAD51B, RAD51C, and XRCC2 (BCDX2 complex), which is regulated by the BRCA1-PALB2-BRCA2 effector complex upstream of RAD51 [3, 4]. Accumulating evidence over the recent years indicates that the inherited inactivation of these HR-associated genes is correlated with a predisposition to female malignancies with various risks and penetrance rates [5]. For instance, *RAD51C* germline variants have been demonstrated to be susceptibility factors for both ovarian and breast cancers. Germline variants of *RAD51D*, most of which are truncated, were principally shown in ovarian cancer with an estimated six-fold increase in risk [6, 7]. Several studies performed in Western and Eastern countries showed evidence of moderate penetrance of ovarian cancer by germline *RAD51D* variants, regardless of ethnicity [8–10]. In a population-based cohort and a screening trial of individuals at high risk of ovarian cancer, both *RAD51C* and *RAD51D* were shown to be moderately susceptible to ovarian cancer, suggesting the usefulness of these genes alongside *BRCA1/2* for the prediction of susceptibility to ovarian cancer [11]. According to another large segregation analysis, the cumulative risks of tubo-ovarian cancer up to the age of 80 years were estimated to be 11 to 13% for *RAD51D* and *RAD51C* variant carriers, respectively [12]. Other epidemiological studies have reported associations between *RAD51D* germline variants and breast cancer risk regardless of the subtype of breast cancer [12, 13]. One study also suggested a potential therapeutic strategy targeting HR-related genes, which show alterations in sarcomas arising from gynecologic organs [14]. The benefits of using poly (ADP-ribose) polymerase (PARP) inhibitors to overcome the very poor prognosis of gynecologic sarcoma have also been shown in a recent study [15]. As further novel evidence, in this case report, we demonstrate a rare peritoneal leiomyosarcoma with a germline background of *RAD51D* c.904-2A > T [NM_002878], a splicing variant of *RAD51D*, that can be interpreted as pathogenic based on its aggressive clinical phenotype in the present case and through computational predictions for attenuated transcription and translation of the gene. We also present a case in which *the RAD51D* variant was identified as a presumed germline pathogenic variant (PGPV) in cancer precision medicine and was successfully referred for genetic counseling including the patient’s family members in a timely manner.

## Case presentation

**Fig. 1:**
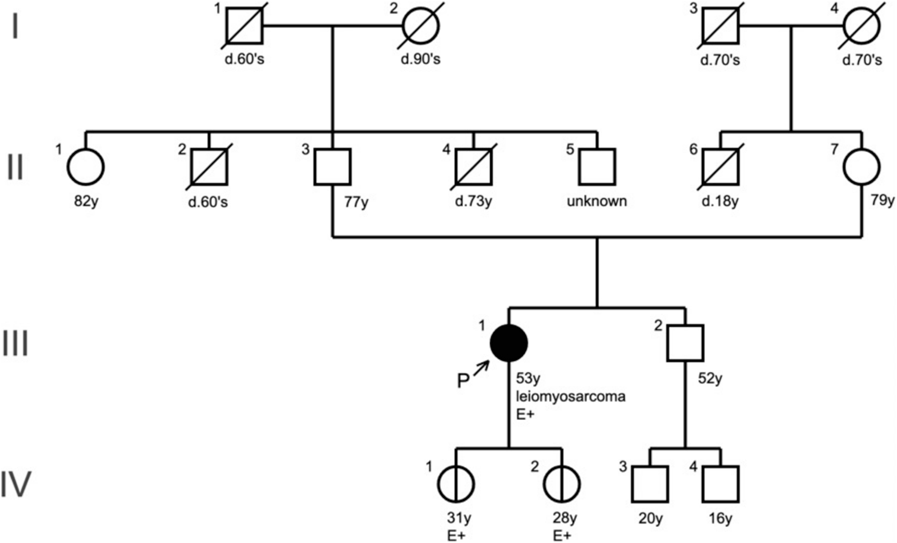
The patient’s family pedigree. The arrow shows the proband (III-1) with leiomyosarcoma. The black circle indicates an individual with cancer. Other symptoms are indicated by unfilled circles or squares. E+ signs (III-1, IV-1 and VI-2) represent a positive evaluation for the indicated variant of *RAD51D* by genetic testing

A Japanese woman in her 50s underwent surgical resection for a tumor arising in the left retroperitoneum. Preoperative computed tomography (CT) and positron emission tomography (PET)-CT as well as intraoperative findings showed no distant metastasis, no obvious peritoneal dissemination, and no invasion of the uterus or ovaries (Supplementary Information). Histologically, the tumor was identified as a leiomyosarcoma of 12 cm × 11 cmin size. She was treated with postoperative chemotherapy with 6 cycles of doxorubicin, followed by chemotherapy with pazopanib for 8 months. During the observational follow-up period, imaging studies indicated peritoneal recurrence. While chemotherapy with pazopanib was promptly restarted, the tumor mass eventually grew to an obvious tumor in the peritoneum. She underwent debulking surgery twice for peritoneal tumors, but the tumors recurred. She received chemotherapy with gemcitabine and docetaxel, which was discontinued due to the onset of interstitial pneumonia. Cancer genome profiling was conducted to search for genome-matched treatment strategies in daily clinical practice in cancer precision medicine. She was referred to us for genetic counseling and interpretation of the *RAD51D* splicing variant, which was originally detected in the tumor genome profiling analysis. She had no family history of RAD51-related cancers or other types of malignancies, including sarcoma (Fig. 1).

**Fig. 2:**
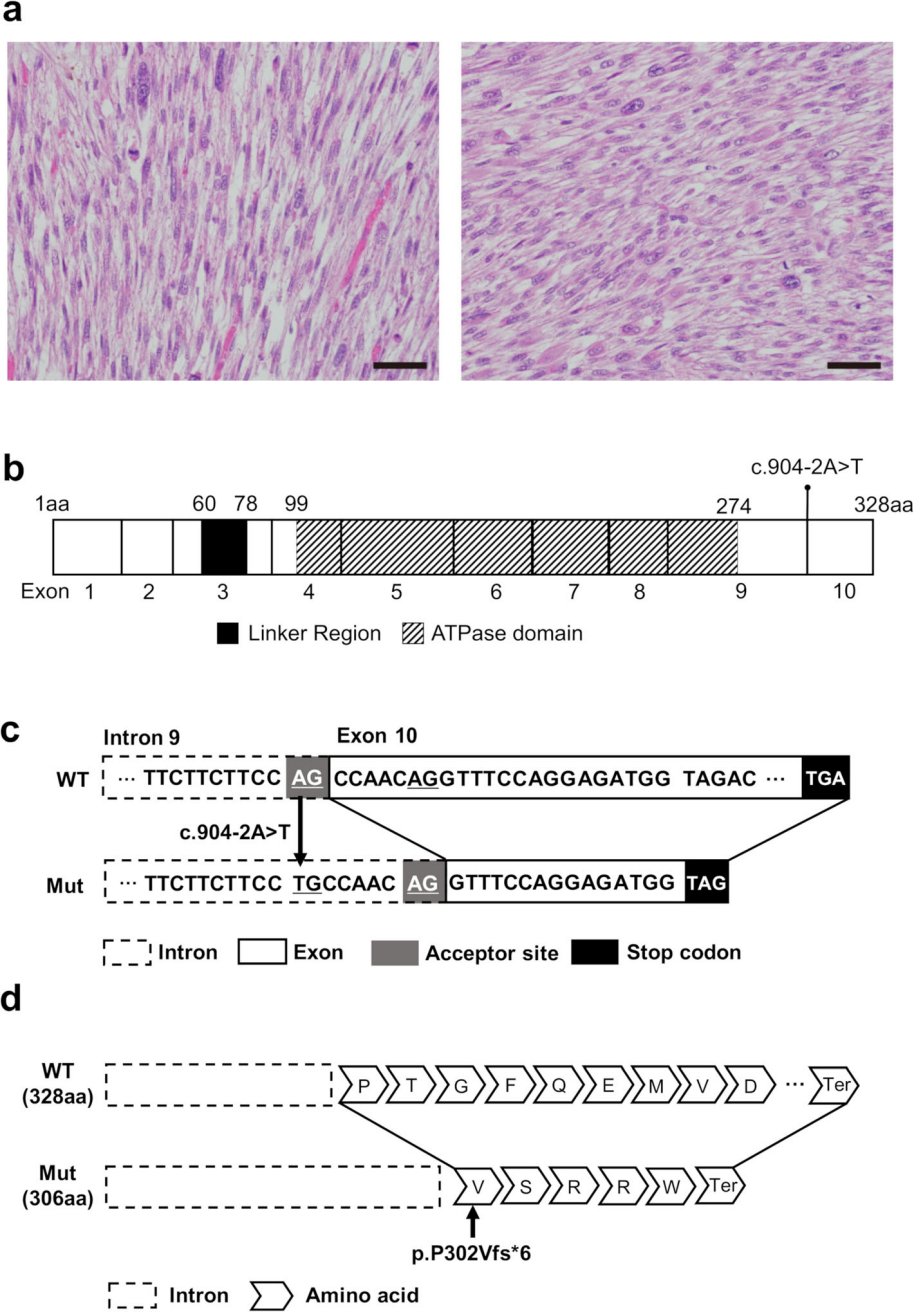
**a** Histological findings of the tumor specimen. Two representative high-magnification views of hematoxylin and eosin-stained specimens are shown. Scale bars = 50 μm. **b** Schematic structure of *RAD51D*. Consensus regions/domains such as linker region and ATPase domain are shown in black or oblique-lined boxes. The number of amino acids (aa 1–328) is displayed on the top of the box. Amino acid numbers for each region/domain were obtained from a previous study by Chen et al. [13]. The number of exons (exons 1–10) translated to proteins are described below the box. The locus of *RAD51D* c.904-2A > T is in the carboxyl terminal of intron 9. Nucleotide numbering was based on the National Cancer for Biotechnology Information (NCBI) (http://www.ncbi.nlm.nih.gov) reference sequence NM_002878. **c** DNA sequences of wild-type (WT) *RAD51D* and the splice variant of *RAD51D* c.904-2A > T (Mut) with a plausible splicing acceptor site in the original exon 10 of *RAD51D*. AGs in gray boxes are splice acceptor sites of WT and an alternative splice site of Mut, respectively. TGA or TAG, outlined in black boxes, are termination codons. We suspect that a new splicing junction appeared 7 bp downstream from the 5′-end of exon 10, which resulted in the production of an aberrant mRNA. **d** Protein structure of WT (328 aa) and a representative variant of Mut (306 aa). The 7-base deletion of exon 10 (c.904_910del) causes a translational frameshift and produces a premature stop codon at six amino acids downstream from the 302nd of valine (abbreviated as V), as shown with an arrow. (p.P302Vfs*6). aa, amino acids; Ter, termination codon (stop codon)

In histological assessments, the tumor showed fascicular proliferation of spindle cells with eosinophilic cytoplasm. The nuclei showed moderate atypia, and some tumor cells had prominent nucleoli. Occasional mitotic figures were also observed. Tumor necrosis was not observed (Fig. 2a). In immunohistochemical studies performed at Mita Hospital adjunctive to the International University of Health and Welfare, Japan, the tumor cells were shown to be weakly positive for α-smooth muscle actin and desmin. These findings are consistent with a diagnosis of leiomyosarcoma, equivalent to histological grade 1 according to the French Federation of Cancer Centers Sarcoma Group (FNCLCC) grading system for sarcoma [16].

Through genomic profiling analysis of the designated 324 genes of the patient’s tumor samples by using the FoundationOne CDx® (Foundation Medicine Inc., MA, USA) oncology panel, two pathogenic variants in *RAD51D* (splicing variant) and *TP53* (missense variant) and intermediate amplifications of four genes were detected (Table 1). On the basis of the recommendations by the expert panel, which is a tumor board that provides molecular and clinical interpretations and suggestions for the results of oncology panel testing, the patient received genetic counseling and subsequently underwent germline analysis for *RAD51D* c.904-2A > T [NM_002878]. The same splicing variant of *RAD51D* was detected in the analysis of DNA extracted from lymphocytes by direct DNA sequencing using capillary gel electrophoresis and fluorescence detection (Sanger sequencing technique and application), which was performed in a Clinical Laboratory Improvement Amendments (CLIA)-certified laboratory. After extensive genetic counseling based on the results obtained from germline testing, her two daughters, shown as IV-1 and IV-2, also underwent predictive genetic testing, and both were found to be positive for the variant (Fig. 1).

**Table 1:** List of genes showing alterations in the tumor-only sequence

Genes	Alteration	CN	VAF (%)
*RAD51D*	Splice site 904-2A>T	N/A	50.99
*TP53*	T155I	N/A	86.63
*AURKB*	Amplification	10	N/A
*C17orf39 (GID4)*	Amplification	9	N/A
*EGFR*	Amplification	7	N/A
*IGF1R*	Amplification	8	N/A

*CN* copy number, *VAF* Variant allele fraction, N/A, not applicable (N/A)

### In silico prediction and clinical interpretation of *RAD51D* c.904-2A > T [NM_002878]

To evaluate the potential effects of the spicing variant due to a single-nucleotide alteration at the 3′-end of intron 9, the schematic structure and location of which are shown in Fig. 2b, we used four computational prediction tools, Max Entropy Scan (MES), NetGene2, Splice Site Prediction by Neural Network (NNSplice), and Alternative Splice Site Predictor (ASSP) to analyze the potential effects of *RAD51D* c.904-2A > T [NM_002878]. All four tools predicted that the *RAD51D* variant attenuated normal splicing and yielded a shorter form of the RAD51D protein (Table 2 and Supplementary Information). Furthermore, cBROCA analysis, an experimental analysis for RNA as shown by Casadei et al. [17], also predicted that this alteration would weaken the native splice acceptor site and create or strengthen alternative novel splice acceptor sites, resulting in shorter forms of the protein products of *RAD51D*. Taken together, these computational analyses suggest that a single-nucleotide alteration in intron 9 of *RAD51D* c.904-2A > T [NM_002878] attenuates normal splicing of *RAD51D* mRNA, resulting in the production of a shorter form of RAD51D protein (a representative truncated product is shown in Fig. 2c and d). Although there are no specific consensus domains in exon 10, which is truncated by attenuated splicing, the aggressively malignant phenotypes observed in this case would be evidence for *RAD51D* c.904-2A > T [NM_002878] as a novel loss-of-function variant involved in the formation and/or progression of malignant tumors.

**Table 2:** *In silico* predictions for the splicing of *RAD51D* wild-type (WT) and c.904-2A>T [NM_002878] (c.904-2A>T)

	WT		c.904-2A>T (Mut)	
Tools	*(A)*	*(B)*	*(A)*	*(B)*
MES	100	29.4	-2.45	137.1
NetGene2	100	163.6	0	165.3
NNSplice	100	-	-	101.1
ASSP	100	67.5	-	89.6

Potential effects on splicing at the original site (*A*) or an alternative site in exon 10 (*B*) predicted by each tool are shown as fold-increase of the prediction score. Raw scores and methods of prediction analysis by each tool and schemas are available in the Supplementary Information

*MES* Max Entropy Scan, *NNSplice* Splice Site Prediction by Neural Network, *ASSP* Alternative Splice Site Predictor

According to the ClinVar database (Variation ID: 472631) (https://www.ncbi.nlm.nih.gov/clinvar/, accessed in August 2021), the clinical significance of *RAD51D* c.904-2A > T [NM_002878] is likely pathogenic or uncertain, meaning that its clinical significance has not yet been determined and is a subject of debate in interpretation. Allele frequencies of *RAD51D* c.904-2A > T (NM_002878) in the general population were 6.60 × 10^− 4^ (ToMMo 8.3KJPN) and 4.13 × 10^− 4^ (HGVD ver2.3, dbSNP rsID: 1403784434) in Japanese people and 3.98 × 10^− 6^ in the global population, suggesting that the splicing variant *RAD51D* c.904-2A > T [NM_002878] was extremely rare in the global population as well as in Japan, which may be potentially indicate that it is a pathogenic variant (Table 3).

**Table 3:** Allele frequencies of *RAD51D* c.904-2A>T [NM_002878] in general population groups

Database	Population	Allele number	Allele frequency
HGVD	Japanese	2,420	4.13 × 10^-4^
GEM-J WGA	Japanese	15,162	6.60 × 10^-4^
ToMMo	Japanese	16,760	6.60 × 10^-4^
KRGDB	Korean	2,922	3.00 × 10^-4^
gnomAD	East Asian	18,394	5.44 × 10^-5^
gnomAD	Global	251,488	3.98 × 10^-6^
TOPMed	Global	264,690	4.00 × 10^-6^

Allele frequency is the fraction with germline variants of *RAD51D* c.904-2A>T among the general population according to each database. Allele number is the total number of allele which was analyzed in each database. All data were obtained by accessing each database in August 2021. Web sites for reference are shown in the Supplementary Information

## Discussion

We report a case presenting with a high implication of pathogenicity for the splicing variant *RAD51D* c.904-2A > T [NM_002878], which was identified through cancer precision medicine in a female patient with retroperitoneal leiomyosarcoma and confirmed to be of germline origin in the patient involved. According to the epidemiological databases of the Genome Aggregation Database (gnomAD) ver 2.1.1 and Trans-Omics for Precision Medicine (TOPMed), the frequencies of *RAD51D* c.904-2A > T [NM_002878] carriers in the general population worldwide are as rare as 3.98 × 10^− 6^ and 4.00 × 10^− 6^, respectively (Table 3). Although the frequencies increased up to 6.60 × 10^− 4^ (ToMMo 8.3KJPN) or 4.13 × 10^− 4^ (HGVD ver2.3) in the general cohorts in Japan, all of them were less than 1 × 10^− 3^ (0.1%), making the variant rare enough to be interpreted as pathogenic, since rarity is a prerequisite factor for the interpretation of clinical pathogenicity of variants according to the practical criteria in clinical genetics [18].

In the previous literature, germline *RAD51D* c.904-2A > T [NM_002878] was reported in a few cases of carcinoma, such as ovarian serous carcinoma, cholangiocarcinoma of the bile duct, and breast carcinoma [19–21] (Table 4). Considering the unfavorable clinical outcomes observed in this patient carrying a germline variant of *RAD51D*, this case provides evidence for the potential pathogenicity of *RAD51D* c.904-2A > T [NM_002878], indicating its susceptibility to a wide range of malignancies. This case shows leiomyosarcoma not only as another related malignancy but also a rare case, which is so-called “off-tumor” [22], in the presence of a germline variant of *RAD51D*.

**Table 4:** Clinical and pathological features of carcinomas with germline *RAD51D* c.904-2A>T reported in the literature

Author, Year	Age, Sex	Organ, Histology	Family history of cancer (Relationship /age of onset)
Our case, 2021	56 y, Female	Peritoneum, Leiomyosarcoma	None
Kaneyasu T, et al., 2020 [19]	42 y, Female	Breast carcinoma	Breast cancer (Maternal aunt/ 50y)
Wardell CP, et al., 2018 [20]	73 y, Male	Biliary tract, Cholangiocarcinoma	None
Eoh KJ, et al., 2018 [21]	58 y, Female	Ovary, Serous carcinoma	None

According to the standards and guidelines for the interpretation of variants recommended by the American College of Medical Genetics and Genomics (ACMG) and the Association for Molecular Pathology (AMP), the presence of intronic variants with loci within ±2 bases from the 5′- or 3′-end of splice sites, as seen in this case, can be rationally interpreted as evidence for pathogenicity because the variants in splicing acceptor sites typically lead to loss of function due to impaired splicing [23]. In silico analyses predicted that *RAD51D* c.904-2A > T [NM_002878] would affect the splice acceptor site of intron 9 and result in frameshifting and attenuated mRNA transcription, with premature stop codons localized in the middle of exon 10 (Fig. 2c and 2d). Analyses using four computational prediction tools supported the higher potential of *RAD51D* c.904-2A > T [NM_002878] than the wild type in the aberrant splicing of *RAD51D* (Table 2).

Although the histological diagnosis for this case using the resected tissue was clearly leiomyosarcoma, there were some tricky points that could otherwise be confused with primary or recurrent peritoneal carcinoma due to the location (peritoneum) and the gene in which the variant was detected (*RAD51D*). Since *RAD51D* is frequently associated with epithelial carcinoma but not with non-epithelial sarcoma, this case provides novel evidence for the crucial role of the *RAD51D* splicing variant in risk elevation for malignancies, including non-epithelial tumors. Another point to be considered in this case was the presence of a variant of *TP53* that was identified in the tumor sequencing (Table 1). Wild-type *TP53* can suppress *RAD51* transcription by binding to the *RAD51* promoter [24]. RAD51 is overexpressed in many tumors as a complementary mechanism against defects in DNA damage repair, including impaired HR by *BRCA1* and *BRCA2* [25]. In addition, *TP53* variants are commonly found in leiomyosarcoma, for example, as many as 50% of sporadic leiomyosarcomas are known to show *TP53* variants [26]. In contrast, the clinical significance of the missense variant of *TP53* (*TP53* T155I) found in tumors in this case is uncertain according to the ClinVar database (Variation ID: 1005876) and neutral in the FATHMM prediction under the COSMIC database (Genomic Mutation ID: COSV52741190) with a score below 0.5 (0.39), suggesting that the *TP53* variant found in this case would be a wild-type-ish variant that would lead to suppression of RAD51 expression due to the regulation mechanism of RAD51 by TP53. Overall, in this case, the non-compensated steady level of RAD51 plus abnormal RAD51D protein production due to its splicing variant might have transformed the tissue phenotypes into a highly aggressive type of sarcoma. Nevertheless, considering the hypothetical nature of these assumptions, they should be analyzed in greater detail in further investigations.

Regarding the family members in this case, two daughters successfully received genetic counseling. The same splicing variant of *RAD51D* was detected in both of them as a germline background (Fig. 1). Their referral to genetic counseling at a proper time point enabled them to start their risk management and clinical surveillance for ovarian cancer, including peritoneal tumor, breast cancer, and even sarcomas. This case is also a good example in which a presumed germline origin variant was found through tumor-only profiling analysis and confirmed by germline testing in a timely manner, even in family members. The clinical surveillance of the family members will provide valuable evidence that will contribute to conclusive determination of the clinical significance of *RAD51D* c.904-2A > T [NM_002878]. By considering an ongoing clinical trial using poly (ADP-ribose) polymerase (PARP) inhibitors on leiomyosarcoma, HR-related genes, including the *RAD51D* splicing variant identified in this case, can be a good clinical molecular marker not only for risk prediction but also for the choice of treatments for leiomyosarcoma.

In conclusion, we present a case of recurrent leiomyosarcoma in the peritoneum that showed evidence of a germline background for the *RAD51D* splicing variant in intron 9 implies the clinical risk elevation for malignant tumors, including sarcomas. To the best of our knowledge, this is the first case report of leiomyosarcoma other than carcinomas with a germline background of *RAD51D* c.904-2A > T [NM_002878].

## Supplementary Information

**Additional file 1.**

## Abbreviations

RAD51D: RAD51 paralog D
HR: Homologous recombination
PGPV: Presumed germline pathogenic variant
FNCLCC: Fédération Nationale des Centres de Lutte Contre le Cancer (in French) French Federation of Cancer Center Sarcoma Group (in English)
CLIA: Clinical Laboratory Improvement Amendments
HGVD: Human genetic variation database
ToMMo: Tohoku Medical Megabank Organization
GEM-J WGA: GEnome Medical (GEM) alliance–Japan Whole Genome Aggregation
KRGDB: Korean reference genome database
gnomAD: The Genome Aggregation Database
TOPMed: Trans-omics for precision medicine
ACMG: American College of Medical Genetics and Genomics
AMP: Association for Molecular Pathology
COSMIC: Catalogue of somatic mutations in cancer
PARP: poly (ADP-ribose) polymerase
