# Supplementary material for: Retroperitoneal leiomyosarcoma in a female patient with a germline splicing variant RAD51D c.904-2A > T: a case report
Source: Hered Cancer Clin Pract. 2021 Nov 27;19:48. doi: 10.1186/s13053-021-00205-x (PMC8627011; doi:10.1186/s13053-021-00205-x)
Supplement: Supplementary file 1 — Additional file 1. [file 13053_2021_205_MOESM1_ESM.zip › supplementary information Futagawa et al_completed_by_authors.pdf]

Futagawa M., et al.

Supplementary Information
Clinical Imaging Data

a

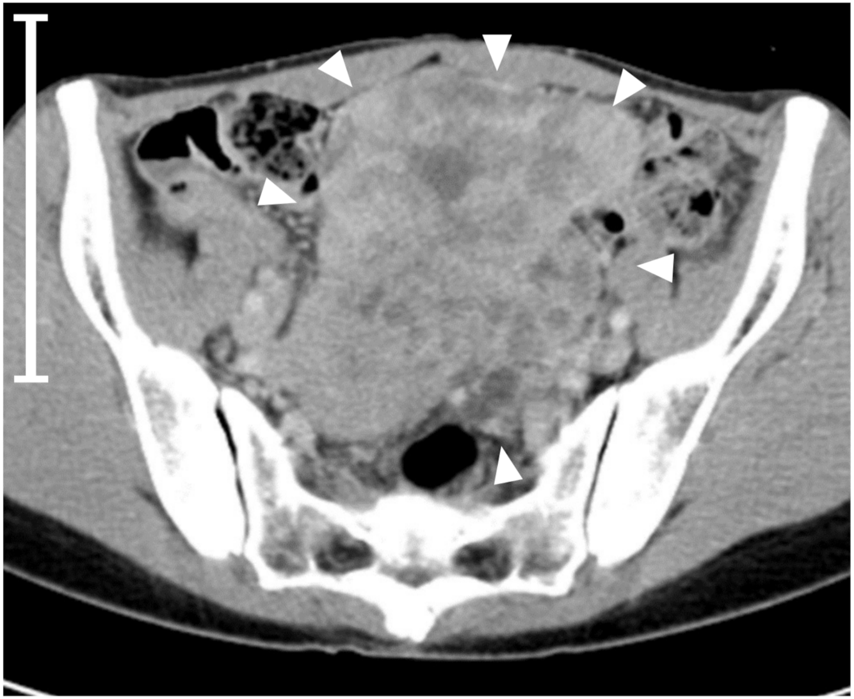

b

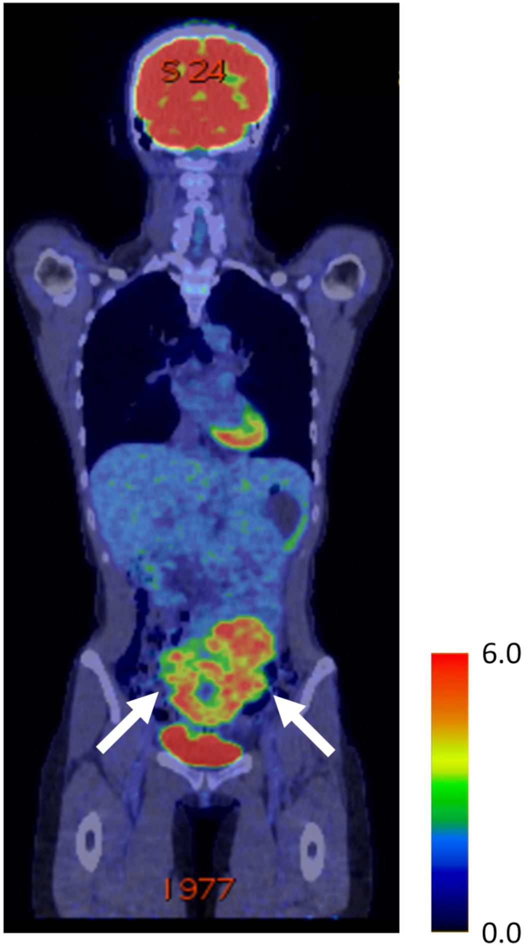

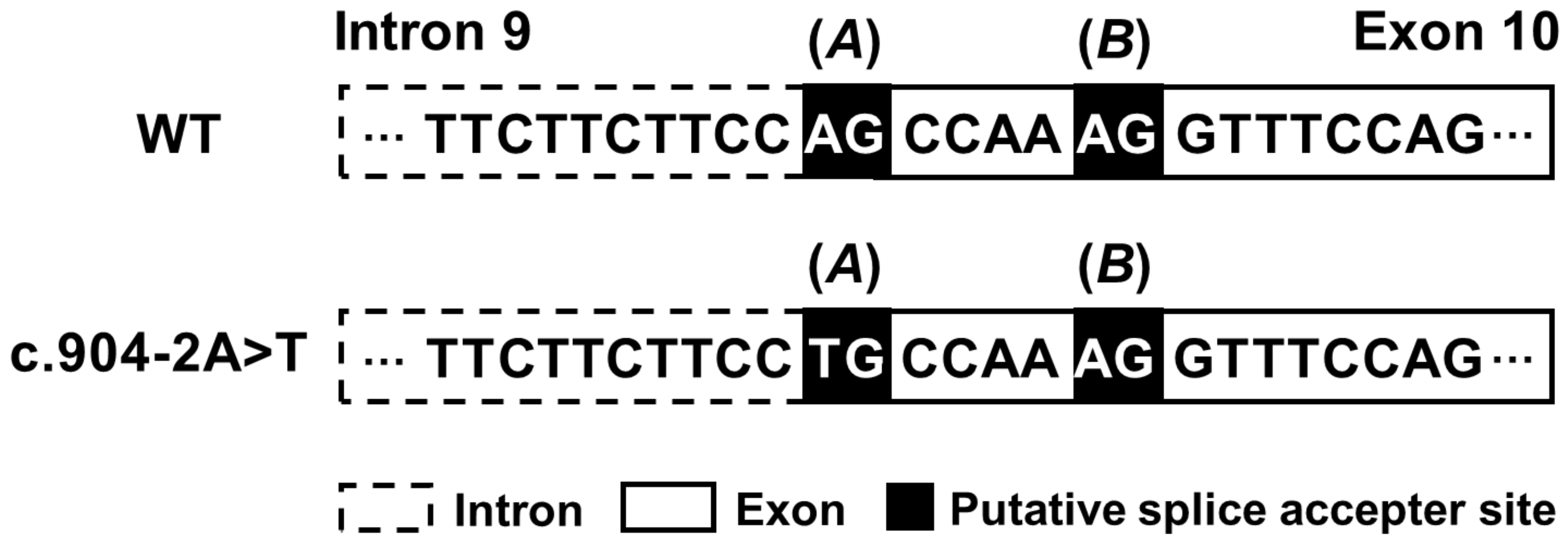

	WT		c.904-2A>T	
	(*A*)	(*B*)	(*A*)	(*B*)
MES	8.16	2.40	-0.20	11.19
NetGene2	0.60	0.982	0.00	0.992
NNSplice	0.95	–	–	0.96
ASSP	10.57	7.138	–	9.47

MES, Max Entropy Scan; NNSplice, Splice Site Prediction by Neural Network; ASSP, Alternative Splice Site Predictor
