# Supplementary material for: Retroperitoneal leiomyosarcoma in a female patient with a germline splicing variant RAD51D c.904-2A > T: a case report
Source: Hered Cancer Clin Pract. 2021 Nov 27;19:48. doi: 10.1186/s13053-021-00205-x (PMC8627011; doi:10.1186/s13053-021-00205-x)
Supplement: Supplementary file 1 — Additional file 1. [file 13053_2021_205_MOESM1_ESM.zip › Supplementary Information_20211109_ESM_final_proofed_by_authors.docx]

***Supplementary Information***

**Clinical imaging data**

**(a)** **Computed tomography (CT) imaging prior to the initial operation.** A solid mass, 12-cm-in-size with irregular margin and partial lobulation was detected as tumor of which main locus is in pelvic lesion. Arrow heads indicate the approximate location of the tumor. Scale bar = 10 cm.

**(b) Positron emission tomography (PET)-CT imaging prior to the initial operation.** Whole body imaging of PET-CT is shown. Standard uptake value (SUV) of the mass in pelvic lesion was calculated as 10.58 maximally. Arrows indicate the location of the tumor. There are no images suggesting for metastasis.

**Supplementary data (figure & table)**

The original acceptor sequence at the 3′-end of intron 9 of wild type (WT) is AG and the variant sequence of the splicing variant (c.904-2A>T) is TG, both of which are displayed in black boxes (***A)***. The alternative putative splice acceptor sites in which the nucleotide sequence AG localized at 7-base-downstream from the original splice acceptor site are displayed in black boxes (***B***). The prediction scores estimating the probability of splicing at the sites of (***A***) or (***B***) in WT and c.904-2A>G were calculated using each predictive tool. The raw prediction scores calculated by each tool are shown in Table.

**Tools for *in silico* analysis**

The following four predictive tools were selected for the *in silico* analysis in this study: MaxEntropy Scan (MES) (http://hollywood.mit.edu/burgelab/maxent/Xmaxentscan_scoreseq_acc.html ), NetGene2 version 2.42 (http://www.cbs.dtu.dk/services/NetGene2/), Splice Site Prediction by Neural Network (NNSplice) version 0.9, (http://www.fruitfly.org/seq_tools/splice.html), and Alternative Splice Site Predictor (ASSP) (http://wangcomputing.com/assp/), all of which are freely available online as of June 2021. Reference genomic sequences of *RAD51D* used for analysis were extracted from the Locus Reference Genomic (LRG) database (NG_031858.1fasta), which consisted of the relevant exonic sequence and 200 bp of flanking intronic sequences.

**Web sites of databases for allele frequency in general population (Table 2)**

The following four databases were used to collect allele frequencies in this study.

Japanese Multi Omics Reference Panel (<https://jmorp.megabank.tohoku.ac.jp/202102/>)

TogoVar (<https://togovar.biosciencedbc.jp/>)

dbSNP (<https://www.ncbi.nlm.nih.gov/snp/>)

gnomAD (<https://gnomad.broadinstitute.org/>)
